# 肺癌患者化疗期间联合营养支持治疗的研究进展

**DOI:** 10.3779/j.issn.1009-3419.2014.12.08

**Published:** 2014-12-20

**Authors:** 艺侨 罗, 江 朱

**Affiliations:** 1 610041 成都，四川大学华西临床医学院 West China Medical Center of Sichuan University, Chengdu 610041, China; 2 610041 成都，四川大学华西医院胸部肿瘤科 Department of Thoracic Oncology, West China Hospital, Sichuan University, Chengdu 610041, China

**Keywords:** 肺肿瘤, 营养支持, 化疗, Lung neoplasms, Nutrition support, Chemotherapy

## Abstract

原发性肺癌是最常见的恶性肿瘤之一，目前其发病率、死亡率均居第一位，在我国仍呈现上升趋势。肺癌患者常常伴有营养不良和消瘦，化疗时细胞毒药物的不良反应可能导致患者营养状况进一步恶化而降低抗肿瘤治疗疗效和患者的生活质量。随着癌症姑息治疗的发展以及患者对生存质量的更高要求，营养支持治疗将成为在化疗过程中维持患者良好的营养状态以及增强自身免疫力的重要辅助治疗手段，在提高肺癌患者化疗耐受性和改善预后等方面发挥积极作用。现就肺癌患者在化疗期间联合应用营养支持治疗的研究进展做一综述。

## Abstract (en)

Primary lung cancer is one of the most common malignancies. Nowadays, both its morbidity and mortality rank first, patients with lung cancer are often goes with some affiliating symptoms such as malnutrition and weight loss. The side effects of cytotoxicity during chemotherapy may lead to further deteriorate of the nutritional status and worsen the anti-tumor therapy's efficacy and the patients' quality of life. With the development of palliative treatment and the higher request of patients for quality of life, nutritional support will be an important adjunctive treatment to maintain a good nutritional status and enhance the patients' immunity during chemotherapy. It will play an active role in improving tolerability of chemotherapy and prognosis for patients with lung cancer. Here is a review about research progress of nutrition support treatment during chemotherapy for the patients with lung cancer.

## 1 前言

目前，肺癌的发病率和死亡率在世界范围内居癌症之首^[1]^，70%-80%的肺癌患者就诊时已失去手术机会，化疗等细胞毒性抗肿瘤治疗仍然是晚期肺癌患者的主要治疗手段。由于癌症的全身性影响，肺癌患者常常伴有一定程度的营养不良，加之细胞毒治疗所引起的不良反应可使患者的营养状况进一步恶化，导致治疗失败，同时降低患者生活质量。因此，有必要对肺癌患者进行营养支持治疗，制止或逆转营养不良持续发展的趋势^[2]^。营养支持可分为肠内营养（enteral nutrition, EN）和肠外营养（parenteral nutrition, PN）。EN是经胃肠道提供代谢需要的营养物质及其他各种营养素的营养支持方式，具有符合人体生理状态、积极促进胃肠道功能恢复以及经济方便的优点^[3]^。目前已发现的并发症有：胃肠道并发症、代谢并发症、感染并发症、精神心理并发症、机械并发症等。肠外营养又分为全肠外营养（total parenteral nutrition, TPN）和部分肠外营养（part parenteral nutrition, PPN），是指从静脉内供给营养素的营养支持方式，该方式对于无法进食进饮的患者适用。但长时间使用肠外营养支持，不符合人体生理，可导致肠粘膜萎缩，影响肠道形态和功能，甚至对免疫系统造成损伤。因此在临床工作中，常根据肺癌患者的实际情况给予肠内或肠外营养支持。2009年，美国肠外肠内营养学会（American Society for Parenteral and Enteral Nutrition, ASPEN）发布了临床肿瘤患者营养支持指南^[4]^，强调没有证据证明营养支持会促进肿瘤生长。这样的结论无疑增强了我们对肺癌患者加强营养支持治疗的信心，同时为我们在肺癌患者接受化疗或同步放化疗期间给予营养支持提供了理论依据。

## 2 营养支持对肺癌患者在化疗期间的有利影响

### 2.1 营养支持对肺癌患者营养状况的影响

晚期肺癌患者常接受含铂联合方案的化疗，由于顺铂等化疗药物的严重消化系统不良反应，导致患者在治疗期间热量和营养素常常摄入不足，尤其是氮的摄入严重缺乏，同时患者在荷瘤状态下产生的炎症因子以及肿瘤细胞本身的副产物都可能导致机体耗能增加、脂肪和骨骼肌的蛋白质分解亢进，尤以骨骼肌消耗明显。故肺癌患者在化疗中或化疗后常常出现比化疗前更为明显的消瘦和营养不良，严重时可出现恶病质。评估肺癌患者营养状况的指标很多，包括体重、皮褶厚度、骨骼肌质量等，生化指标有血浆总蛋白、白蛋白和前白蛋白等，在临床上常联合应用上述指标根据实际情况来判定肺癌患者的营养状况。Murphy等^[5]^将接受化疗的非小细胞肺癌（non-small cell lung cancer, NSCLC）患者分为肠内营养支持组和标准治疗组，利用测量体重、骨骼肌质量以及皮下脂肪组织等指标来比较两组患者化疗期间的营养状况，结果显示：NSCLC患者化疗期间肠内营养的介入能够维持患者体重和骨骼肌质量，改善营养不良的状况。另一项研究^[6]^将40例Ⅲ期的NSCLC患者随机分为干预组[给予高能口服营养补充剂含二十碳五烯酸（eicosapntemacnioc acid, EPA）2.02 g/d及二十二碳六烯酸（docosahexaenoic acid, DHA）0.92 g/d]和对照组(给予等热量的营养剂但不含EPA和DHA)，结果干预组患者的体重维持情况优于对照组。高小荃等^[7]^将120例NSCLC患者分为干预组（在放化疗过程中给予肠内及肠外营养支持）和对照组（给予常规治疗），发现干预组患者的营养状况评分、体脂肪、腰围、臀围等指标均优于对照组，作者认为给予适当的营养支持能够有效改善NSCLC患者的营养状况，增强其机体功能，对于肺癌的治疗具有积极意义。李红晨等^[8]^的研究发现，在化疗期间接受肠外营养支持治疗的肺癌患者的血清总蛋白、白蛋白、前白蛋白及转铁蛋白等营养相关指标均高于对照组。唐冉冉等^[9]^将60例包含肺癌的恶性肿瘤患者分为实验组和对照组，实验组在常规饮食的基础上，化疗期间联合应用肠内营养支持，而对照组仅给予常规饮食，在第二周期化疗结束后，研究组患者的营养状况未见明显变化，但对照组患者则明显下降，提示在反复化疗损伤的环境下，肠内营养或许能避免化疗导致的消化道反应影响，为机体提供热能和营养，且效果明显。以上多个临床研究均体现肺癌患者在化疗期间应用营养支持治疗能够改善营养状况，提高患者的化疗的耐受能力，帮助肺癌患者更安全地渡过化疗期。

### 2.2 营养支持对肺癌患者免疫功能的影响

肺癌患者的免疫状态与肿瘤的发生、发展以及各个方面有密切联系。由于现有的化疗药物特异性不强，在杀灭肿瘤细胞的同时常常伴随着对自身免疫系统的抑制；再加上肺癌患者通常伴随着较长期的营养不良^[10, 11]^，上述两个因素的综合作用，可以导致肺癌患者免疫系统功能的紊乱，患者的治疗耐受力进一步减弱，同时也为肿瘤进展埋下了伏笔。化疗后患者的外周血总淋巴细胞计数、CD3^+^、CD4^+^、CD8^+^和NK细胞计数均明显减少。肺癌患者上述指标水平被认为与分期有关，在早、中期患者的水平往往偏高，而晚期尤其是发生转移的患者则明显降低^[12]^。化疗所致的免疫抑制主要表现为T细胞减少。因此T细胞亚群是监测化疗期间肺癌患者免疫功能改变的重要指标。李红晨等^[13]^将132例肺癌患者分为对照组（63例）和观察组（69例），在化疗过程中，对照组给予普通饮食，观察组给予肠外营养支持，研究发现化疗后观察组白细胞计数及血小板计数均较对照组明显增高，同时两组NK细胞计数、CD3^+^、CD3^+^CD4^+^及CD4^+^/CD8^+^差异均有统计学意义。提示在肺癌患者化疗期间同时予以营养支持可能使患者较快恢复免疫功能，有一定的细胞免疫调节作用。

另一方面，由B细胞介导的体液免疫，能产生IgG、IgM等免疫球蛋白，体液免疫通过补体依赖性细胞毒性作用（complement dependent cytotoxicity, CDC）、抗体依赖性细胞介导的细胞毒作用（antibody dependent cell mediated cytotoxicity, ADCC)和增强肿瘤免疫反应等机制发挥抗肿瘤作用^[14]^。在肺癌患者化疗过程中，免疫球蛋白IgG及IgM随化疗的进行而持续下降。可能与化疗期间B细胞被大量杀伤有关。甘燕青等^[15]^将肺癌术后120例患者随机分为肠内营养组（60例）以及胃肠外营养组（60例）探讨肺癌术后患者采用哪种方式能够更快地促进患者的免疫系统恢复。结果显示，通过肠内营养方式支持治疗的患者，免疫球蛋白水平上升的幅度明显高于肠外支持治疗。可见肠内营养治疗对于肺癌术后患者体液免疫功能恢复有积极的作用。

### 2.3 营养支持对抗肿瘤治疗的影响

#### 2.3.1 营养支持提高化疗的完成率

化疗是肺癌治疗中的重要手段，但由于化疗药物多具有较强的毒副作用，部分患者常难以耐受^[16]^。特别是老年肺癌患者常常伴有营养不良、抵抗力下降等，在机体一般情况较差的情况下进行化疗可能会进一步破坏机体的免疫功能，有些患者甚至不能耐受化疗而停止治疗^[17]^。Ross等^[18]^通过对780例包括肺癌在内的肿瘤患者进行对比研究发现：在NSCLC患者中，营养不良的患者较体重正常的患者化疗副反应更为明显，且更容易出现化疗延迟。万涛等^[19]^随机选取了70例肺癌化疗的患者并分为研究组和对照组，对照组患者的饮食按照日常习惯进行，研究组患者额外给予肠外营养支持治疗，对患者化疗后的临床表现进行观察与比较发现，在白细胞计数的下降率、化疗依从性以及不良反应发生率等方面，研究组明显优于对照组，提示营养支持或许能够降低感染率、提高治疗完成率以及减少化疗造成的不良反应。

#### 2.3.2 营养支持与化疗疗效

由于营养不良、血浆蛋白水平降低，机体对化疗药物的吸收、分布、代谢及排泄均产生障碍，明显影响了化疗药物的药代动力学，导致化疗药物的毒性增加，机体耐受性下降，抗肿瘤疗效也受到明显影响^[20]^。Huhmann等^[21]^总结多项研究后得出结论：积极营养支持治疗可以使原本无法接受化疗的恶性肿瘤患者重新获得化疗机会，认为将抗肿瘤治疗与营养支持治疗二者联合有益于患者生存期延长。孙士玲等^[22]^通过回顾性分析老年肺癌患者91例的临床资料，依据患者化疗期间是否接受肠内营养支持分为研究组（43例），给予化疗及肠内营养支持；对照组（48例），给予化疗及普通饮食。发现研究组化疗期间并发症发生率明显低于对照组（*P*=0.038）；研究组化疗后2年生存率明显高于对照组（*P*=0.030）。营养支持或许可以提高肺癌患者对化疗的耐受性，增加依从性，从而改善化疗疗效。

### 2.4 营养支持对肺癌患者生活质量的影响

肺癌患者的生活质量评价常常采用欧洲癌症研究与治疗组织（European Organzation for Research and Treatment of Cancer, EORTC）的生活质量核心问卷（QLQ-C30）和肺癌特异性模块QLQ-LCl3来评价。Van Der Meij等^[23]^对n-3多不饱和脂肪酸在肺癌患者营养支持中的作用进行随机双盲对照研究，发现干预组在生活质量参数、身体及认知功能方面都比对照组有更好的表现，提示补充n-3多不饱和脂肪酸或许能够使肺癌患者在整体生活质量方面获益。崔灵芝等^[24]^通过对100例在化疗期间的恶性肿瘤患者（随机分为观察组与对照组，观察组给予部分肠外营养支持，对照组不给于肠外营养支持）以问卷方式对患者生活质量（qulity of life, QOL）进行评分，通过对化疗开始后第8、15、22、29、36、43天的QQL评分进行比较，发现观察组整体的QOL评分高于对照组。提示积极给予肠外营养支持可以改善患者机体的一般状况，提高生存质量。刘美玲等^[25]^探讨了老年NSCLC患者接受化疗的同时补充氨基酸对其生活质量的影响，将74例老年NSCLC患者随机分为试验组与对照组，试验组化疗同时补充9复合氨基酸（9-AA）500 mL/d，对照组常规化疗。结果显示化疗后试验组患者在躯体、情绪和社会功能等方面均较对照组有明显优势；失眠、疲倦、口腔疼痛和食欲下降等化疗副反应较对照组轻，患者的生活质量得到有效改善。以上研究结果均提示在肺癌患者接受化疗期间给予肠内或肠外营养支持能够有效的降低治疗过程中引起的副反应和并发症，提高患者对化疗的耐受能力，并且能够在很大程度上起到心理抚慰作用，改善患者心境，从而提高肺癌患者在化疗过程中及化疗后的生活质量。

### 2.5 关于肺癌患者应用营养支持的相关指南与量表

目前，欧美国家也正逐渐加大对肿瘤营养学研究的投入，以期在肿瘤的综合治疗中发挥营养支持治疗的重要作用。2009年，美国肠外肠内营养学会发布了肿瘤患者营养支持治疗的新指南，强调在进行积极的抗肿瘤治疗的患者中，如果存在营养不良或有营养不良的风险时，进行营养支持能改善患者的营养状况，提高免疫功能^[26]^。欧洲肠外肠内营养学会（Guidelines on Parenteral Nutrition: Non-surgical oncology, ESPEN）也提供了恶性肿瘤患者尤其是放化疗患者及恶病质患者的营养支持治疗指南，供我国肿瘤医师作诊疗参考。中国临床肿瘤学会（Chinese Society of Clinical Oncology, CSCO）肿瘤营养治疗专家委员会在2011年得出专家共识，也为我国肿瘤患者的营养支持治疗提供了参考。即便如此，肿瘤患者中营养不良的发生率仍然高达40%-80%。如何早期发现营养不良成为一个关键性的问题，这就要求我们重视营养不良的筛查与评估。目前临床使用可以进行营养不良筛查的量表有患者主观整体评估量表（Patient-Generated Subjective Global Assessment, PG-SGA）、总体主观量表（SGA）、营养风险筛查量表-2002（nutritional risk screening-2002, NS-2002）等，其中PG-SGA专门为肿瘤患者所设计，简便而准确，是一个比较适合肿瘤患者进行营养风险筛查的量表，值得在临床上推广和应用。

## 3 总结与展望

本文综述了肺癌患者在化疗期间应用营养支持治疗的优势：联合营养支持能够在一定程度上改善患者的营养状况、免疫机能、生活质量，减少化疗的不良反应，提高化疗完成率和疗效。因而肺癌患者在化疗期间联合营养支持治疗是必要的，具有推广价值。

同时应该看到，作为姑息治疗的重要内容之一，营养支持治疗在肺癌化疗患者中的临床研究还明显不足，亟需我们进行更加深入而全面的探讨。
